# A Retrospective Observational Study on the Eleganza Protocol: A Standardized Seven-Step Workflow for Combined Aesthetic Surgery

**DOI:** 10.7759/cureus.113875

**Published:** 2026-08-03

**Authors:** Francisco A Rodríguez-García, Raul E Melo-Ventura, Héctor Álvarez-Trejo, Félix O Márquez-Villalobos, Emmanuel Dorado-Hernández, Gerardo García-Dávila, Andrea P Camarena-Macias, Quitzia L Torres-Salazar

**Affiliations:** 1 Plastic and Reconstructive Surgery, Eleganza Plastic Surgery Center, Zamora, MEX; 2 Plastic and Reconstructive Surgery, Eleganza Plastic Surgery Center, Tijuana, MEX; 3 Biomedical Sciences, Directorate of Teaching, Quality, and Research, Servicios de Salud de Durango, Durango, MEX

**Keywords:** abdominoplasty, body contouring, combined, liposuction, mammoplasty, operative time, surgical procedures

## Abstract

Background

Combined aesthetic surgery ("mommy makeover") is increasingly performed to address postpartum body contour deformities; however, prolonged operative time remains associated with increased perioperative morbidity. The Eleganza Protocol was developed as a standardized workflow-oriented approach designed to optimize operative sequencing and operative efficiency during combined aesthetic surgery.

Methods

A retrospective observational study was conducted including 13 consecutive patients treated at two independent surgical centers using the Eleganza Protocol. The protocol consists of a predefined seven-step operative sequence integrating coordinated team-based execution, neuraxial anesthesia, and an anterior-to-posterior surgical workflow. Combined procedures included mastopexy, breast augmentation, abdominoplasty, and extensive liposuction. Primary outcomes were operative time and 30-day postoperative complications classified according to the Clavien-Dindo system. Secondary outcomes included estimated blood loss and intraoperative transfusion requirements.

Results

Most procedures were completed within four hours, with nine of 13 patients (69.2%) finishing within this threshold (median operative time: 3.5 hours; q25-q75: 3.0-4.5). The median estimated blood loss was 250 mL (q25-q75: 100-300 mL), and no intraoperative transfusions were required. No major postoperative complications (Clavien-Dindo grade ≥II) occurred during the 30-day follow-up period. Minor self-limited complications included transient edema and ecchymosis. No patient required prolonged hospitalization beyond the planned overnight stay.

Conclusions

This retrospective observational study describes operative performance and short-term safety in consecutive patients undergoing combined aesthetic surgery using the Eleganza Protocol. Operative times remained within a consistent range despite procedural variability, with low estimated blood loss and no major short-term postoperative complications. Further prospective multicenter studies are warranted to evaluate reproducibility, operative performance, safety, and long-term outcomes.

## Introduction

In recent years, the demand for combined aesthetic surgical procedures has increased significantly, particularly among postpartum women seeking comprehensive body rejuvenation. The so-called "mommy makeover" has emerged as a popular surgical package that typically includes mammoplasty, abdominoplasty, and regional liposuction. This approach addresses multiple anatomical changes resulting from pregnancy, aging, and weight fluctuations in a single operative session, offering patients the benefit of a unified recovery period and potentially improved aesthetic harmony [1,2]. In this study, the term "mommy makeover" is used descriptively to reflect its widespread clinical use rather than a formal procedural classification.

However, combining procedures inherently increases operative complexity and duration, often exceeding four hours [2]. Prolonged surgical time correlates with greater morbidity, including higher risk of venous thromboembolism, infection, and transfusion [3]. Systematic reviews report odds ratios of 5.35-14.71 for major complications when abdominoplasty is combined with breast surgery versus abdominoplasty alone [1]. Likewise, Shermak underscores that optimizing intraoperative efficiency (reducing hypothermia and blood loss) is key to minimizing perioperative risk [4].

While multiple surgical techniques for combined aesthetic procedures have been described, most focus primarily on anatomical correction and aesthetic outcomes. In contrast, limited attention has been given to standardized and reproducible surgical workflows designed to optimize operative sequencing and time efficiency without compromising safety. As a result, considerable variability persists in operative duration, team coordination, and perioperative management across institutions and surgeons.

To address this methodological gap, we developed the Eleganza Protocol, a standardized, workflow-oriented surgical method designed to enable the completion of comprehensive mommy makeover procedures within a predictable operative timeframe. The protocol integrates a seven-step surgical sequence, coordinated team-based execution, neuraxial anesthesia, and an anterior-to-posterior operative strategy aimed at minimizing repositioning, blood loss, and unnecessary delays.

The primary objective of this study was to describe the operative performance of the Eleganza Protocol in consecutive patients undergoing combined aesthetic surgery. Secondary objectives were to report perioperative outcomes and short-term safety during the 30-day postoperative period. This report also presents the protocol’s structure and its implementation in patients treated at two independent surgical centers. The study is reported in accordance with the Strengthening the Reporting of Observational Studies in Epidemiology (STROBE) Statement guidelines [5].

## Materials and methods

This retrospective observational study included consecutive female patients who underwent combined aesthetic surgery using the Eleganza Protocol between 2024 and 2025 at Vitangeli Surgical Center in Zamora, Michoacán, Mexico, and NewCity Hospital in Tijuana, Baja California, Mexico, two certified short-stay hospitals with hospital-based operating rooms. All consecutive eligible patients treated during the study period were included using a total population sampling approach; therefore, no formal sample size calculation was performed. Patients were routinely hospitalized overnight and discharged the following day. All procedures were performed by the same fixed surgical team, consisting of a board-certified plastic surgeon as the lead surgeon, an anesthesiologist, a first assistant, and a scrub nurse. Team composition and assigned roles remained constant across cases to reduce workflow variability. Procedures were conducted under standardized operative and anesthetic conditions according to a predefined institutional workflow. Inclusion criteria comprised women aged 18-60 years with body mass index (BMI) <35 kg/m² undergoing combined procedures including breast surgery (augmentation and/or mastopexy), abdominoplasty, and regional liposuction. Patients were classified as American Society of Anesthesiologists (ASA) physical status I or II. Exclusion criteria included significant cardiovascular disease, diabetes mellitus, coagulopathies, active smoking within six months, previous extensive abdominal surgery, and contraindications to neuraxial anesthesia.

All patients underwent preoperative clinical evaluation, photographic documentation, and individualized surgical planning. Anatomical markings were performed in the upright position. Procedures were carried out by a fixed four-member team consisting of one lead plastic surgeon, one anesthesiologist, one first assistant, and one scrub nurse throughout the study period.

All procedures were performed in certified hospital-based operating rooms using standard warming systems and warmed intravenous fluids. Liposuction was performed using surgeon-selected power-assisted or suction-assisted systems with 3-5 mm cannulas according to anatomical requirements. Fat grafting was performed using closed-system collection and blunt-tip cannulas (3-4 mm). Abdominoplasty employed standard electrosurgical instruments, barbed and braided sutures for rectus plication, absorbable layered closure, and closed-suction drains. When indicated, silicone implants were selected according to patient anatomy.

All procedures were performed under standardized neuraxial anesthesia with intravenous sedation and spontaneous ventilation. A combined neuraxial block using hyperbaric bupivacaine and epidural analgesia was performed, followed by bilateral ultrasound-guided transversus abdominis plane blocks at procedure completion. Neuraxial anesthesia with intravenous sedation was selected by the surgical team because it was considered compatible with the objectives of the protocol, including maintenance of spontaneous ventilation, effective perioperative analgesia, rapid postoperative recovery, and reduced exposure to general anesthetic agents. This approach was also considered potentially beneficial for limiting postoperative opioid requirements, nausea, and prolonged recovery. Venous thromboembolism risk was assessed using the Caprini score. Mechanical prophylaxis consisted of compression stockings and intermittent pneumatic compression during surgery, followed by early postoperative ambulation. Pharmacological thromboprophylaxis was not administered in this cohort. Prophylactic cefazolin, 1 g intravenously, was administered 30-60 minutes before surgery. Perioperative analgesic and antiemetic regimens followed institutional protocols.

The Eleganza Protocol

Breast Surgery

**Figure 1:**
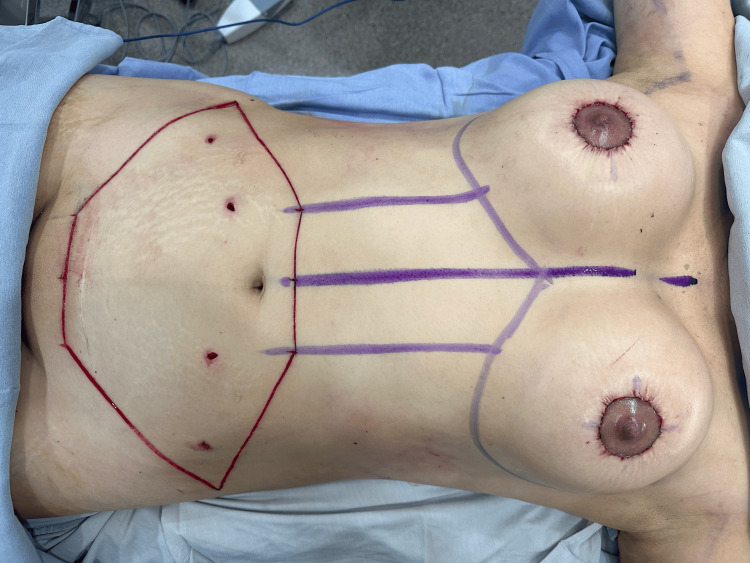
Intraoperative view after the completion of mastopexy with implant placement and before abdominal flap resection, showing final breast contour and pre-marked abdominal resection lines

Breast augmentation was performed through a 3-4 cm inframammary incision using subfascial or submuscular implant placement according to patient anatomy. Mastopexy markings followed the planned pattern (periareolar, vertical, inverted-T, or L). When combined with augmentation, implant placement preceded skin resection. Layered closure was performed with absorbable sutures (Figure 1).

Liposuction

Tumescent infiltration included tranexamic acid and epinephrine for bleeding control. Liposuction of the abdomen, back, arms, and inner thighs was performed using power-assisted or suction-assisted techniques according to anatomical requirements. Harvested fat was processed in a closed system for subsequent gluteal grafting. Aspirate volumes were calculated according to previously published safety parameters.

Abdominoplasty

**Figure 2:**
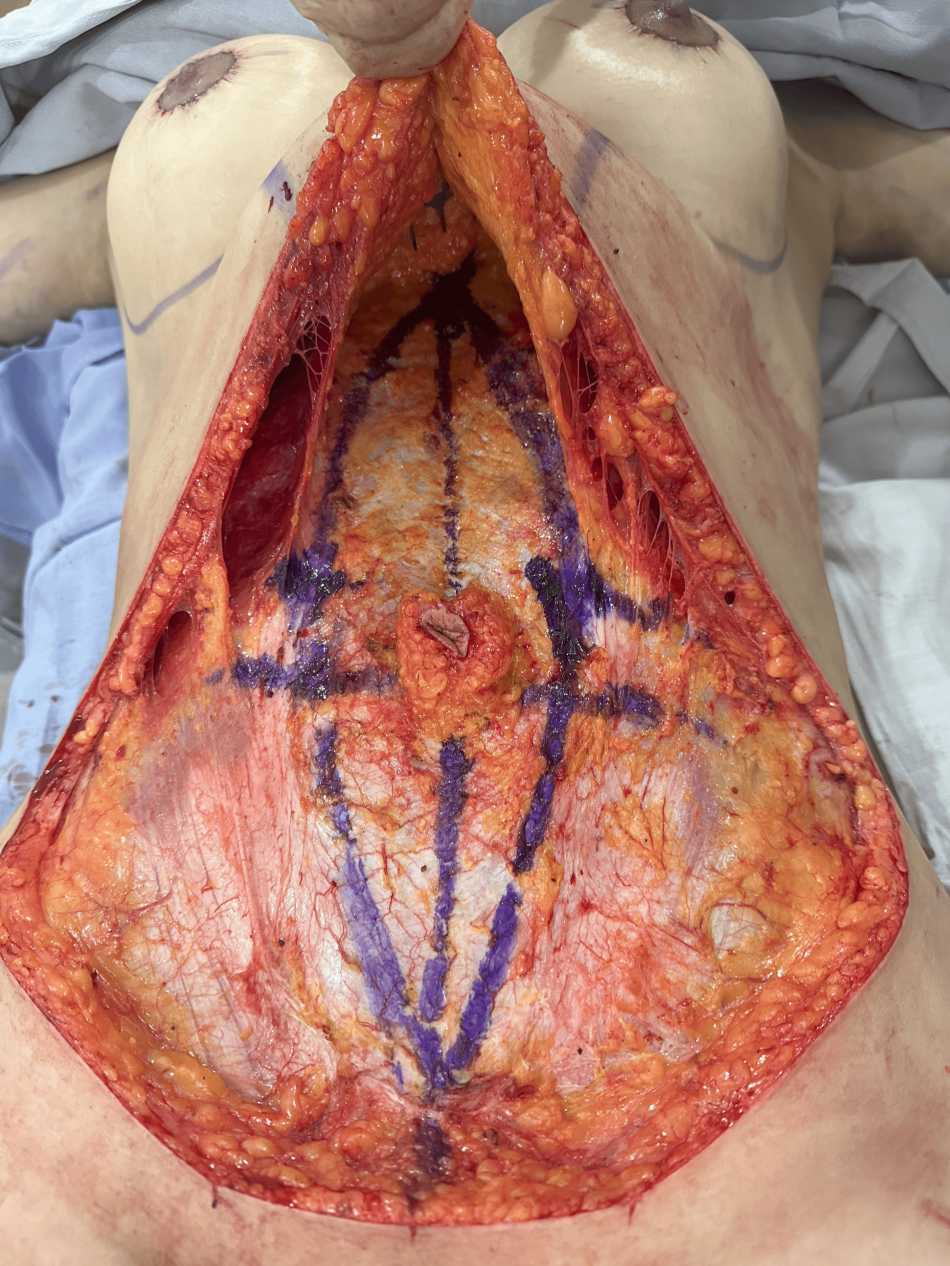
Intraoperative view during anterior abdominoplasty The abdominal flap has been elevated, exposing the rectus abdominis muscles and marked diastasis for subsequent plication.

A low transverse incision was performed with flap dissection to the xiphoid while preserving a periumbilical vascular halo. Rectus diastasis repair, umbilical repositioning, drain placement, and layered closure were performed using standard techniques (Figure 2). Extended and circumferential variants followed the same operative principles.

Combined procedure workflow

The Eleganza Protocol is based on a predefined anterior-to-posterior workflow intended to minimize repositioning and maintain continuous operative progression. Protective cushions were routinely used during prone positioning to avoid pressure on recently operated areas.

Step 1

Asepsis, antisepsis, and tumescent infiltration of the abdomen, lateral torso, arms, and inner thighs were performed with the patient in the supine position (5-10 minutes). The infiltration and latency period were included within the total operative time. During this interval, other surgical steps were performed concurrently.

Step 2

Breast surgery was performed during the tumescent latency period (80-90 minutes). While dissection and hemostasis of the second breast were completed, the assistant simultaneously performed layered closure of the first breast, maintaining uninterrupted operative progression.

Step 3

Liposuction of the lateral abdomen, torso, arms, and thighs was initiated approximately 30 minutes after infiltration and proceeded concurrently with the completion of breast closure (25-30 minutes).

Step 4

Anterior abdominoplasty was initiated approximately one hour after the initial incision and continued concurrently with the final stages of breast closure (50-60 minutes).

Step 5

**Figure 3:**
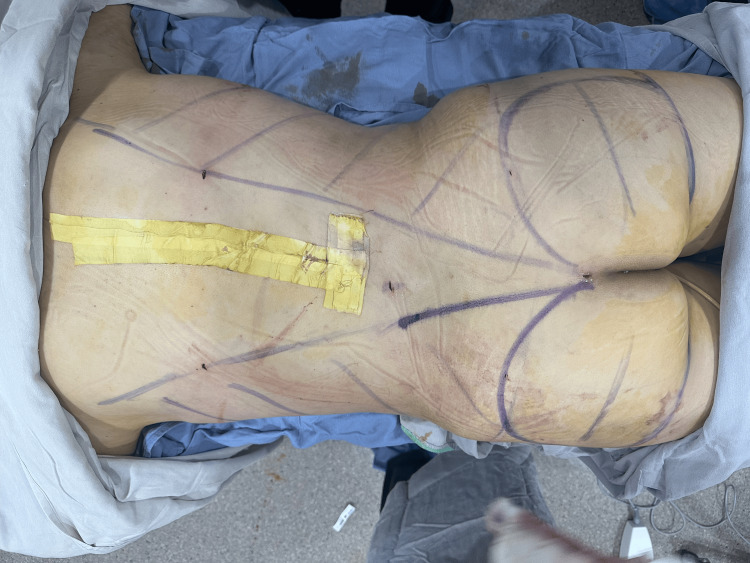
Intraoperative view of the posterior torso during prone positioning Power-assisted liposuction of the back and safe-zone gluteal fat grafting markings are shown.

After the completion of the anterior surgical stage, the patient was repositioned from the supine to the prone position. Posterior torso infiltration and liposuction were then performed (20-30 minutes) (Figure 3). Completion of all anterior procedures before repositioning allowed a single-position change during the operation. Protective cushions were routinely used during prone positioning to avoid pressure on recently sutured areas.

Step 6

Gluteal fat grafting was performed using a 4 mm blunt cannula with a single lateral orifice introduced through intergluteal and gluteal-fold access sites. Fat was injected into the subcutaneous and subfascial planes, treating the gluteal region as a continuous anatomical unit. The average graft volume ranged from 250 to 400 mL per buttock according to individual morphology. Ultrasound guidance was not employed. Fat placement followed established anatomical safety principles intended to avoid deep intramuscular injection (10-15 minutes).

Step 7

Circumferential abdominoplasty, when indicated, was completed after posterior liposuction (30-45 minutes).

Demographic, operative, and postoperative variables were extracted from anonymized medical records. Postoperative follow-up was performed by the operating surgical team according to the institution's standardized postoperative care protocol through the 30-day follow-up period. Closed-suction Blake or Jackson-Pratt drains were placed according to the procedures performed. Drain removal was generally considered between postoperative days 7 and 10 when the output remained at or below 30 mL per day for three consecutive days.

Recorded variables included age, BMI, procedures performed, type of abdominoplasty, operative time, estimated blood loss, intraoperative transfusion requirement, postoperative complications classified according to the Clavien-Dindo system [6], postoperative nausea or vomiting, urinary retention, transient intraoperative hypotension requiring treatment, and postoperative pain assessed using the visual analog scale (VAS) [7]. Operative time and 30-day postoperative complications were considered the primary study outcomes, whereas estimated blood loss, intraoperative transfusion requirement, perioperative adverse events, and postoperative pain were evaluated as secondary outcomes. Operative time was defined as the interval from the administration of the neuraxial block to the completion of the procedure, including skin closure and application of the simple postoperative dressings. It also included anesthetic block latency, tumescent infiltration and latency, all applicable surgical steps, and intraoperative patient repositioning. Recovery time and transfer from the operating room were not included.

Continuous variables were summarized as medians and 25th-75th percentiles because of the small sample size. Categorical variables were expressed as frequencies and percentages. Given the descriptive objective of the study and the absence of a comparison group, no inferential statistical analyses were performed. Statistical analyses were conducted using IBM SPSS Statistics for Windows, V. 27.0 (IBM Corp., Armonk, NY, USA).

## Results

A total of 13 consecutive patients were included in the study. The median age was 40 years (q25-q75: 34-43), and the median BMI was 29.4 kg/m² (q25-q75: 28-31). All patients had completed childbearing before surgery. The median number of procedures performed during each operative session was four (q25-q75: 4-5). A history of cesarean section was documented in eight patients (61.5%). All 13 patients (100%) underwent mastopexy with T-pattern skin resection. The closure pattern (inverted-T or vertical) was individualized according to breast morphology and the degree of ptosis. Abdominoplasty and liposuction of the abdomen and back were performed in 13 patients (100%). Breast augmentation and gluteal fat grafting were each performed in 10 patients (76.9%), whereas arm liposuction was performed in five patients (38.5%). Less frequently performed adjunctive procedures included cruroplasty, rib remodeling, and J-Plasma skin tightening, each performed in one patient (7.7%), adding approximately 10-15 minutes to the operative time (Tables 1-2).

**Table 1:** Baseline characteristics of the study population *Continuous variables are presented as median (25th-75th percentiles) and minimum-maximum (N=13). BMI: body mass index

Variable	Value	Minimum-maximum
Age (years)*	40 (34-43)	27-59
BMI (kg/m²)*	29.4 (28-31)	27.5-32.1
Parity*	3 (2-3)	2-3
Previous cesarean section	8 (61.5%)	-
Number of procedures per operative session*	4 (4-5)	3-6
Estimated blood loss (mL)*	250 (100-300)	100-350

**Table 2:** Surgical procedures of the study population

Procedures performed	n (%)
Mastopexy with T-pattern resection	13 (100%)
Breast augmentation	10 (76.9%)
Liposuction of the abdomen and back	13 (100%)
Arm liposuction	5 (38.5%)
Abdominoplasty	13 (100%)
Gluteal fat grafting	10 (76.9%)
Cruroplasty	1 (7.7%)
Costal remodeling	1 (7.7%)
J-Plasma skin tightening	1 (7.7%)

**Figure 4:**
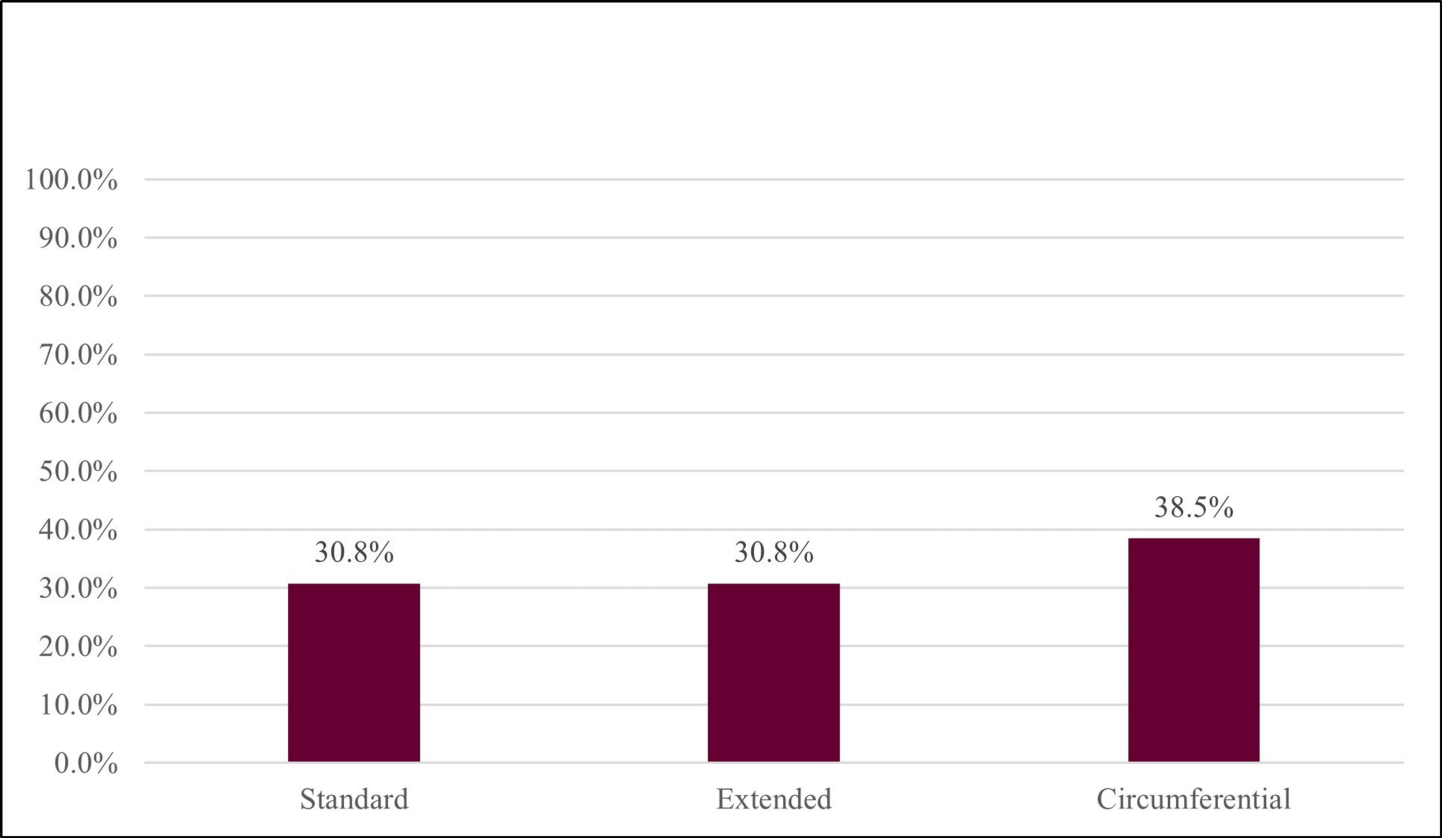
Type of abdominoplasty performed in the study population

Regarding the type of abdominoplasty, four patients (30.8%) underwent standard abdominoplasty, four (30.8%) underwent extended abdominoplasty, and five (38.5%) underwent circumferential abdominoplasty (Figure 4).

**Figure 5:**
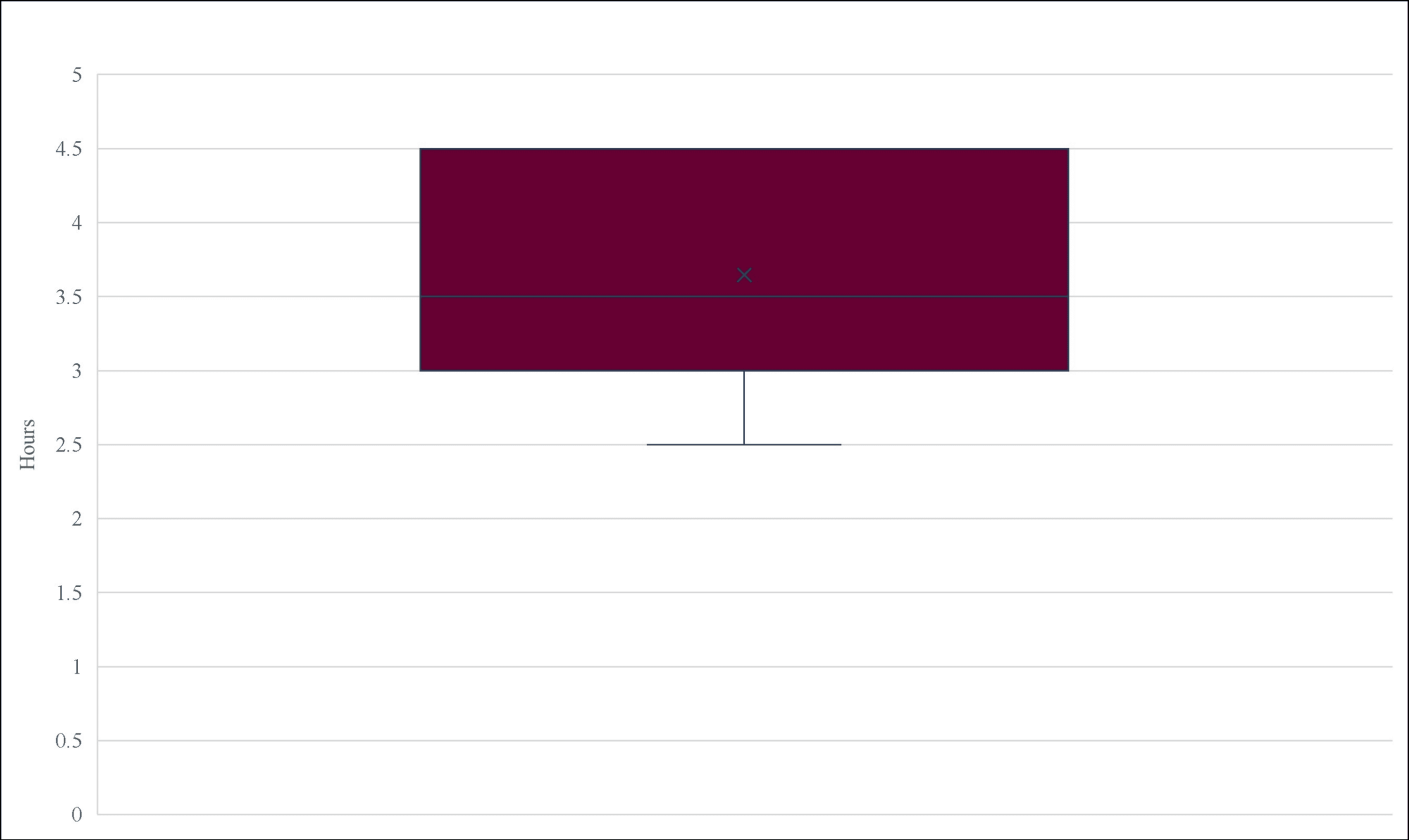
Distribution of operative time in the study population

The median operative time was 3.5 hours (q25-q75: 3.0-4.5; minimum-maximum: 2.5-4.5). Nine procedures (69.2%) were completed within four hours, and no operation exceeded 4.5 hours (Figure 5).

The median estimated blood loss was 250 mL (q25-q75: 100-300 mL), and no patient required intraoperative blood transfusion. No patient experienced postoperative urinary retention, nausea, or vomiting. Transient intraoperative hypotension requiring intravenous fluid administration occurred in one patient (7.7%), with no subsequent clinical consequences. Postoperative pain remained below 6 points on the VAS throughout follow-up.

No patient developed a major postoperative complication (Clavien-Dindo grade II or higher) during the 30-day follow-up period. One patient (7.7%) developed a localized mildly hypertrophic suprapubic scar with hyperpigmentation, which improved with conservative topical management. Localized edema and transient ecchymosis were each observed in one patient (7.7%), and both resolved spontaneously without additional intervention.

## Discussion

The Eleganza Protocol was developed to address the inherent challenges of combined aesthetic surgery, particularly "mommy makeover" procedures, which are frequently associated with prolonged operative times and increased perioperative risk. Large series such as the CosmetAssure analysis by Gupta et al. demonstrated that combined breast and abdominoplasty procedures present higher complication rates than isolated procedures, particularly regarding hematoma, infection, and venous thromboembolism [8]. Operative duration and BMI remain important factors in perioperative risk stratification.

In the present study, mastopexy, breast augmentation, abdominoplasty, and extensive liposuction were completed with a median operative time of 3.5 hours, with most procedures performed within four hours. For contextual reference, multicenter series describing isolated lipoabdominoplasty procedures have reported operative times approaching 3.9 hours [9]. Although direct comparisons cannot be established due to differences in study design and procedural complexity, these findings provide contextual reference for the operative time profile observed in the present study.

Interpretation of operative duration should consider the heterogeneity of procedures included. Although the Eleganza Protocol standardized the operative sequence, the combination of surgical components varied according to individual patient requirements, including breast augmentation, gluteal fat grafting, and adjunctive contouring procedures. Despite this variability, the same anterior-to-posterior workflow was consistently applied in all cases.

The protocol incorporates structured operative sequencing, coordinated team-based execution, and integration of simultaneous surgical steps intended to maintain continuous operative progression throughout the procedure. Sequential body-contouring procedures may substantially prolong total operative duration, particularly when multiple procedures are combined during the same session [10,11]. In a retrospective analysis of 1,753 plastic surgery cases, Hardy et al. documented substantial variability in operative duration across surgical categories, with median operative times ranging from 2.38 hours among cases classified as "other breast surgery" to 8.77 hours among cases involving autologous breast reconstruction; 75.8% of the operations included combined procedures, with an average of 4.9 concurrent procedures [11]. In a separate study, Alvarez-Trejo et al. reported that intramuscular gluteal augmentation alone required approximately 60-100 minutes, depending on the technique employed [10]. In this context, operative workflow organization becomes particularly relevant in combined procedures.

Perioperative optimization represented an important component of patient selection and operative planning within this study. Preoperative hemoglobin levels were generally optimized to values above 12-14 g/dL, although individualized assessment allowed lower thresholds in selected patients depending on procedural extent and overall clinical condition. In addition, our group previously reported the use of tranexamic acid within the tumescent infiltration solution and a quantitative formula for calculating safe aspirate volumes considering both liposuction and flap resection [12]. These perioperative measures may have contributed to the estimated blood loss and absence of transfusion observed in this study; however, these observations should be interpreted descriptively within the limitations of a retrospective observational study.

Because the objective of this study was descriptive, the present findings should not be interpreted as evidence that the Eleganza Protocol shortens operative time or reduces complications compared with other surgical workflows. Rather, they describe the operative performance observed during the implementation of a standardized workflow in consecutive patients.

Shermak emphasized that operative times exceeding six hours are associated with increased complication risk in combined aesthetic surgery [4]. In the present study, all procedures remained below this threshold, and no major postoperative complications were observed during short-term follow-up. Throughout the study, mechanical thromboprophylaxis, early ambulation, neuraxial anesthesia, and standardized perioperative management were systematically implemented as part of the institutional protocol.

A distinctive feature of the Eleganza Protocol is its predefined seven-step workflow integrating simultaneous operative tasks within a structured and standardized surgical sequence. Rather than introducing a novel isolated surgical maneuver, the protocol focuses on operative organization, procedural coordination, and allowing continuous operative progression during complex combined aesthetic surgery. The present study also highlights the importance of strict patient selection and perioperative optimization when performing combined body-contouring procedures.

Previous reports have suggested that combined body-contouring procedures may be performed with acceptable short-term safety profiles when careful patient selection, meticulous operative planning, and standardized perioperative management are applied. Shermak emphasized that abdominoplasty combined with breast or contouring procedures does not necessarily increase complication rates when these principles are respected [4]. Similarly, contemporary series such as those reported by Cortes et al. described acceptable short-term safety in procedures involving circumferential liposuction, abdominoplasty, and fat grafting [13]. In the present study, no Clavien-Dindo grade II or higher complications were observed, and only minor self-limited postoperative complications occurred during short-term follow-up. Mechanical thromboprophylaxis, early ambulation, neuraxial anesthesia, and a standardized operative workflow were maintained throughout the study; however, these observations should be interpreted descriptively given the retrospective observational design and limited sample size.

This study has several limitations. It reflects the experience of two specialized surgical centers and lacks a control group, limiting external generalizability. Quantitative follow-up was restricted to 30 days, precluding the evaluation of long-term complications, scar maturation, revision rates, and patient-reported outcomes, including formal assessment of satisfaction. Although some patients continued clinical follow-up for up to six months, photographic documentation is presented for illustrative purposes only. In addition, all procedures were performed by an experienced and well-coordinated surgical team, which may influence reproducibility in other clinical settings. As emphasized by Shermak, combined aesthetic surgery is not appropriate for all patients, and comorbidities such as obesity, diabetes, or active smoking may require staged procedures or hospital-based perioperative management [4].

Despite these limitations, this study provides a structured description of the Eleganza Protocol and its implementation in combined aesthetic surgery. Future prospective multicenter studies are warranted to evaluate reproducibility, operative performance, and short- and long-term safety outcomes in broader patient populations.

## Conclusions

The Eleganza Protocol provides a standardized workflow for combined aesthetic surgery based on a predefined seven-step operative sequence. This retrospective observational study describes operative performance and short-term safety in consecutive patients undergoing combined aesthetic surgery using the Eleganza Protocol. Operative times remained within a consistent range despite procedural variability, and no major postoperative complications were observed during the 30-day follow-up period. These findings suggest that this standardized operative workflow is feasible in appropriately selected patients within the clinical settings evaluated. Further prospective multicenter studies are warranted to evaluate reproducibility, operative performance, safety, and long-term outcomes.
